# Vitamin D Deficiency in Turkish Mothers and Their Neonates and in Women of Reproductive Age

**DOI:** 10.4274/jcrpe.v1i6.266

**Published:** 2010-12-08

**Authors:** Ayça Törel Ergür, Merih Berberoğlu, Begüm Atasay, Zeynep Şıklar, Pelin Bilir, Saadet Arsan, Feride Söylemez, Gönül Öcal

**Affiliations:** 1 Ankara University, School of Medicine, Departments of Pediatric Endocrinology, Ankara, Turkey; 2 Ankara University, School of Medicine, Departments of Neonatology, Ankara, Turkey; 3 Ankara University, School of Medicine, Departments of Gynecology and Obstetrics, Ankara, Turkey; +90 312 204 40 00 - 312 204 41 70+90 533 691 76 28aycaergur@superonline.comAnkara University, School of Medicine, Departments of Pediatric Endocrinology, Ankara, Turkey

**Keywords:** 25OHD, newborn, mother, cord, reproductive age women

## Abstract

**Objective**: Materno-fetal vitamin D deficiency (VDD) may occur in the early neonatal period. We aimed to evaluate the vitamin D (vitD) status and risk factors for VDD in healthy newborns and their mothers, and also in fertile women.

**Methods**: Serum 25 hydroxyvitamin D3 (25(OH)D), calcium (Ca), phosphorus (P) and alkaline phosphatase (ALP) levels were measured in 70 mothers (study group) and their newborns, and in umbilical cord samples. 104 nonpregnant fertile women comprised the control group. Demographic factors such as education and clothing habits of the mother, number of pregnancies and month of delivery were recorded. A serum 25(OH)D level below 11 ng/ml was accepted as severe, 11-25 ng/ml as moderate VDD, and a value over 25ng/ml as normal.

**Results**: Severe VDD was found in 27% of the mothers, and moderate deficiency in 54.3%. Severe VDD was detected in 64.3% of the neonates, and moderate deficiency in 32.9%. Only 18.6% of the mothers and 2.9 % of the neonates had normal vitD levels. In thecontrol group, severe VDD was observed in 26.9%, and moderatedeficiency in 45.2 %. Only 27.8 % of the controls had normal vitD levels. In the control group, the 25(OH)D levels of the women dressed in modern clothes were significantly higher than those of the women wearing traditional clothes. This difference was not observed in the study group because 75% of these 70 mothers wore modern clothes. Mothers giving birth during the summer months and their neonates had significantly higher serum 25(OH)D levels than those of the mothers giving birth during the winter months and their neonates.

**Conclusion**: The study has shown that in Turkey VDD is an important problem in women of reproductive age, in mothers and their neonates. The 25(OH)D levels obtained from the cord may serve as a guide in the determination of the high risk groups.

**Conflict of interest:**None declared.

## INTRODUCTION

Materno-fetal vitamin D deficiency (VDD) is still an important cause of morbidity in developing countries. VDD rickets occurs most commonly during infancy, starting in the early months of life. 25-hydroxyvitamin D3 (25(OH)D) crossing the placenta during the last months of gestation furnishes the main vitamin D (vitD) requirement of the newborn during the first few months of life (1). It is well known that the human milk content of vitD is very low (2). The vitD status of breastfed newborns depends mainly on the vitD stores acquired during intrauterine life (3). VDD rickets in early infancy is prevalent in infants of mothers who have poor vitD stores (4). Turkey is a country, which, in most of its geographic regions, has sufficient sunshine to maintain adequate vitD status by dermal synthesis. However, most Turkish women of reproductive age cannot benefit from this source because of their clothing habits (5).

In this study, we aimed to find out the factors affecting vitD levels by determining the vitD status of women of reproductive age, as well as that of mothers and their offspring. In addition, we investigated the interrelations between calcium (Ca), phosphorus (P), alkaline phosphatase (ALP) and 25(OH)D levels during the first 24 hours after delivery in the mothers, in the umbilical cord and in the neonates. Besides emphasizing this important public health issue, in this preliminary study, we aimed to suggest a method to estimate the risk for VDD in newborns.

## METHODS

This study was carried out from July 2003 through May 2005 in the Department of Pediatric Endocrinology of the Faculty of Medicine, Ankara University. The inclusion criteria of the study are shown in Table 1. 

The study included 104 nonpregnant fertile women in the control group, and 70 mothers and their 70 neonates in the study group. None of the women or of the neonates in this study had previously received vitD supplementation. A detailed history was obtained from each woman including age, number of pregnancies, educational status, and type of clothing (traditional clothes in Turkey cover the hair, arms and legs completely). Month of delivery was also recorded. The blood samples from the newborns were taken within four days after delivery.

The 25(OH)D levels were determined chromatographically, using the isocratic HPLC system (6). Severe VDD was defined as a serum 25(OH)D level below 11 ng/ml. Serum 25(OH)D levels between 11-25 ng/ml were accepted as moderate VDD, and levels above 25 ng/ml were considered to be normal (1).

**Statistical Analysis**

SPSS (Inc Version 10 software) statistical programs were used to analyse the results. The results are expressed as mean and median values, and as a percentage in qualitative variables. The distribution of variables was analysed with the Kolmogorov-Simirnov test. The differences were assessed by Student’s t test or Mann-Whitney nonparametric U test as appropriate. Other statistical evaluations, namely, one-way Anova test, Pearson chi-square test, variance analysis, and Bonferroni test were also used. Statistical significance was considered as p<0.05. No statistical evaluation of the impact of education or prematurity could be made because of similar educational levels and the paucity of preterm infants in the study group.

**Table 1:**
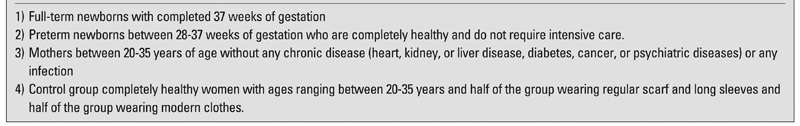
Criteria for inclusion to the study

## RESULTS

The mean age of the mothers in the study group was 29.7±4.7 years and that of the women in the control group was 29.7±4.3 years. The mean gestational age of the neonates was 38.2±2.1 weeks. Eighty percent of the neonates were term and 20% preterm. Mean number of deliveries of the mothers in the study group was 1.7±0.9 and that of the control group was 1.5±1.2 (Table 2).

**Figure 1:**
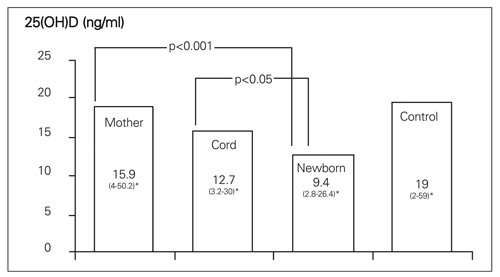
Median serum 25(OH)D levels of all the cases *range of values

The median serum 25(OH)D levels in the mothers, in the umbilical cord, newborns, and in control women are shown in Figure 1. The newborns displayed a lower serum 25(OH)D level than those of mothers. Cord 25(OH)D levels were found to be between the respective levels of the mothers and the neonates. There was no difference in the median serum 25(OH)D level between preterm and term neonates. 

In 33.3% of mothers and 7.1% of neonates, hypocalcemia was encountered. The median serum calcium (Ca) level of the hypocalcemic newborns was 1.95 (1.75-2) mmol/L and all of them presented with symptoms of hypocalcemia such as irritability and seizures. In 3 mothers (13.6%), serum Ca levels were low, while serum 25(OH)D levels were normal. While the serum ALP levels of the mothers were significantly high as compared to those of the controls, the serum Ca levels were found to be significantly low (Table 3). 

Severe VDD was detected in 27% of the mothers and moderate deficiency in 54.3%. Only 18.6% of the mothers had normal vitD levels. Severe and moderate VDD were found in 64.3% and 32.9% of the neonates, respectively. Only 2.9% of the neonates had normal vitD levels. In the control group, severe and moderate VDD were found to be 26.9% and 45.2%, respectively. There were normal vitD levels in only 27.8% of the controls (Table 4). In the control group, the 25(OH)D levels of cases wearing traditional clothes were lower than those of the cases dressed in modern clothes: 13.5 ng/ml; 22.8 ng/ml, respectively (p<0.05). Mothers giving birth during the summer months and their neonates had significantly higher serum 25(OH)D levels than the mothers giving birth during the winter months, 18.1 ng/ml, 10.1 ng/ml, 13.9 ng/ml and 7.9 ng/ml, respectively (p<0.05).

**Table 2:**
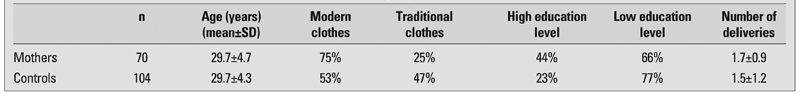
Characteristics of the mothers and of the women in the control group

**Table 3:**
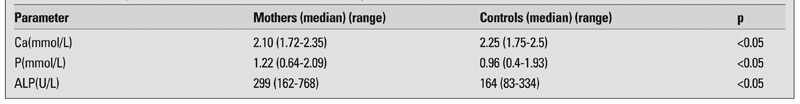
Biochemical parameters in the mothers and in the control group

**Table 4:**
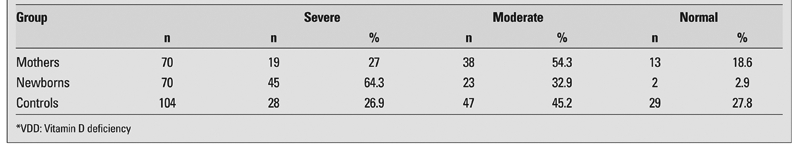
Distribution of VDD*

## DISCUSSION

In this study, 81.4% of the mothers had serum 25(OH)D concentrations in the VDD range (severe-moderate deficiency) and serum 25(OH)D levels were below 25 ng/ml in 97.2% of their neonates. These findings suggest that maternal VDD is the most important factor for VDD in newborns. In a study in Pakistan, more than half of VDD cases were diagnosed in neonates of mothers with VDD (7). In a previous study conducted in Turkey, VDD was found in 54% of the mothers and 80% of the neonates (5). Taha et al. studied 100 Saudi Arabian mothers and their neonates, and they observed severe VDD in 59% of the mothers and 70% of the newborns (8). Bassir et al reported VDD in 80% of the neonates of 50 mothers after delivery (9). Our results are in agreement with all these reports. 

There was no difference in the median serum 25(OH)D level between preterm and term neonates; however, the number of preterm neonates in our study was low.

Although the serum 25(OH)D levels in 13.6% of mothers were normal, the low Ca levels suggested the possibility of nutritional hypocalcemic rickets.Unfortunately, our study did not include an assessment of Ca intake of the mothers. In comparison to investigations made in Africa, it was established that the ratio of nutritional hypocalcemic rickets was lower in our country (10). The findings of high serum ALP and low serum Ca levels in mothers as compared with the control group are probably due to physiological changes related to pregnancy (11).

In this study, the umbilical cord 25(OH)D levels were found to be between those of the mothers and of the neonates. Our findings indicate cord vitD levels may, as a noninvasive method, be an important tool in determining the risk groups for VDD. Routine cord 25(OH)D measurements may be recommended in mothers wearing traditional clothes that cover their bodies completely.

In the control group of 104 nonpregnant fertile women, 49 women wearing traditional clothes had lower 25(OH)D levels than those who wear modern clothes. This difference was not observed in the study group because 75% of the mothers were women dressed in modern clothes. However, this group of mothers represented a middle socioeconomic class of urban women who did not have a lifestyle that would expose them to sunshine. Also, there is a possibility that they may carry a specific ethnical polymorphism in their vitD gene which makes them prone to VDD. Thus, one can say that not only the clothing habits, but also daily lifestyles can make a difference. In a study from Nigeria, it was found that the 25(OH)D levels of mothers wearing veils were significantly lower than the women not wearing veils (12). In another study performed in Oslo, the 25(OH)D levels in Pakistani mothers were significantly lower than those of Norwegian mothers (13). 

In our study, we observed that season of delivery affected significantly the 25(OH)D levels of both mothers and neonates. This correlation has also been found in other studies in Turkey (14, 15). In both of these studies, serum 25(OH)D levels in pregnant women giving birth in summer and their neonates were reported to be significantly higher than those of women giving birth in winter and of their neonates. Moya et al. determined significantly high 25(OH)D levels in 23 healthy mothers and neonates born in summer when compared to those in winter (16). Madelenat et al recommended administration of 80000 IU vitD to pregnant women who were between the 27^th^ and 32^nd^ weeks of their pregnancy during winter months. After this treatment had been administered to 59 pregnant women, no signs of vitD overdose or intoxication in any baby or mother were observed, and the neonates were protected against VDD (17).

In Turkey, a country where VDD continues to be a public health problem, we believe that vitD prophylaxis targeted to all women of reproductive age might be a safe approach. Cord blood sampling during delivery is a non-invasive procedure and may be used as a tool to estimate the risk for VDD in the newborn and to plan a prophylactic approach to the mother aiming to decrease the morbidity of the subsequent offspring. Although we think that screening for VDD by cord blood sampling during delivery is an appropriate method, cost analysis should be evaluated precisely before making any recommendations. At present, we do not have any data for the cost of morbidities arising from materno-fetal VDD in children or in women. However, this issue is far beyond the scope of this pilot study, the aim of which was to emphasize this important public health issue and to make recommendations for considering an alternative feasible evaluation method. We think cord blood 25(OH)D levels can be used to screen VDD. 

In conclusion, this study aimed to demonstrate thefrequency of the VDD problem observed among women of reproductive age, as well as among mothers and their offspring. We advise that all fertile women should be considered candidates for vitD prophylaxis. In addition, the determination of the 25(OH)D levels in the blood sample obtained from the cord was shown to be a no-ninvasive method in determining the risk groups.

## References

[1] Hochberg Z, Bereket A, Davenport M, Delemarre-Van de Waal HA, De Schepper J, Levine MA, Shaw N, Schoenau E, van Coeverden SC, Weisman Y, Zadik Z (2002). Consensus Development for the Supplementation of Vitamin D in Childhood and Adolescence.. Horm Res.

[2] Reese LE, Chesney RW, De Luca HF (1982). Vitamin D of human milk identification of biologically active form.. Am J Clin Nutr.

[3] Fraser DR (1995). Vitamin D.. Lancet.

[4] Akpede GO, Omotara BA, Ambe JP (1999). Rickets and deprivation: a Nigerian study.. J R Soc Promot Health.

[5] Andıran N, Yordam N, Özon A (2002). Risk factors for vitamin D Deficiency in Breast fed newborns and their mothers.. Nutrition.

[6] Holick MF, DeLuca HF (1971). A new chromatographic system for vitamin D3 and its metabolites: resoluation of a new vitamin D3 metabolite.. J Lipid Res.

[7] Atiq M, Suria A, Nizami SQ, Ahmed I (1998). Vitamin D status of breastfed Pakistani infants.. Acta Paediatr.

[8] Taha SA, Dost SM, Sedrani SH (1984). 25 Hydroxivitamin D and total calcium: extraordinarily low plasma concentrations in Saudi mothers and their neonates.. Pediatr Res.

[9] Bassir M, Laborie S, Lapillonne A, Claris O, Chappuis MC, Salle BL (2001). Vitamin D deficiency in Iranian mothers and their neonates: a pilot study.. Acta Paediatr.

[10] Fischer PR, Rahman A, Cimma JP, Kyaw-Myint TO, Kabir AR, Talukder K, Hassan N, Manaster BJ, Staab DB, Duxbury JM, Welch RM, Meisner CA, Haque S, Combs GF Jr (1999). Nutritional rickets without vitamin D deficiency in Bangladesh.. J Trop Pediatr.

[11] Zeni SN, Ortela Soler CR, Lazzari A, López L, Suarez M, Di Gregorio S, Somoza JI, de Portela ML (2003). Interrelationship between bone turnover markers and dietary calcium intake in pregnant women: a longitudinal study.. Bone.

[12] Okonufua F, Houlder S, Bell J, Dandona P (1986). Vitamin D nutrition in pregnant Nigerian women at term and their newborns infants.. J Clin Pathol.

[13] Brunvand L, Hauge E (1993). Vitamin D deficiency amongst Pakistani women in Oslo.. Acta Obstet Gynecol Scand.

[14] Hasanoğlu A, Özalp İ, Özsoylu Ş (1981). Anne ve kordon kanında 25OHD değerleri.. Çocuk Sağlığı ve Hastalıkları Dergisi.

[15] Aydın A, Ilıkkan B, Haktan M, Kavunoğlu G (1988). Doğum sırasındaki annelerdeki D vit düzeyleri ve bu düzeylerin mevsimlerle ilişkisi..

[16] Moya M, Doménech E, López-Arias C, Calzadilla CH, Barroso F, Gonzâlez-Espinosa C, Rodrîguez-Luis JC, Alvarez J (1982). Seasonal variation and diagnostic value of plasmatic levels of 25-hydroxycholecalciferol.. An Esp Pediatr.

[17] Madelenat P, Bastian H, Menn S (2001). [Winter supplementation in the 3rd trimester of pregnancy by a dose of 80,000 IU of vitamin D].. J Gynecol Obstet Biol Reprod (Paris).

